# The Clinical Characteristics and Outcomes of Adult Patients With Pneumonia Related to Three Paramyxoviruses

**DOI:** 10.3389/fmed.2020.574128

**Published:** 2021-01-18

**Authors:** Liang Chen, Xiudi Han, YanLi Li, Chunxiao Zhang, Xiqian Xing

**Affiliations:** ^1^Department of Infectious Diseases, Beijing Jishuitan Hospital, 4th Medical College of Peking University, Beijing, China; ^2^Department of Pulmonary and Critical Care Medicine, Qingdao Municipal Hospital, Qingdao, China; ^3^Department of Infectious Diseases and Clinical Microbiology, Beijing Chao-Yang Hospital, Capital Medical University, Beijing, China; ^4^Department of Pulmonary and Critical Care Medicine, Beijing Huimin Hospital, Beijing, China; ^5^Department of Pulmonary and Critical Care Medicine, The 2nd People's Hospital of Yunnan Province, Kunming, China

**Keywords:** paramyxovirus, pneumonia, clinical characteritic, disease severity, adult

## Abstract

**Background:** Respiratory syncytial virus (RSV), human metapneumovirus (hMPV), and human parainfluenza virus (hPIV) are paramyxoviruses (PMVs) that are important etiologies of community-acquired pneumonia. However, current knowledge about the clinical features and outcomes of PMV-related pneumonia (PMV-p) is limited. We aimed to investigate the clinical characteristics and disease severity in immunocompetent adults hospitalized with hMPV-related pneumonia (hMPV-p), hPIV-related pneumonia (hPIV-p), or RSV-related pneumonia (RSV-p).

**Methods:** We retrospectively recruited 488 patients with PMV-p (153 with RSV-p, 137 with hMPV-p, and 198 with hPIV-p) from five teaching hospitals in China during 2011–2019. Univariate and multivariate analyses were performed to identify predictors to distinguish hMPV-p/hPIV-p from RSV-p and evaluate the effects of virus types on the clinical outcomes.

**Results:** Compared with RSV-p, sputum production [*odds ratio* (*OR*) 5.029, *95% confidence interval* (*CI*) 2.452–10.312, *P* < 0.001] was positively associated with hMPV-p, while solid malignant tumor (*OR* 0.346, *95% CI* 0.126–0.945, *P* = 0.038), nasal congestion (*OR* 0.102, *95% CI* 0.041–0.251, *P* < 0.001), and respiratory rate ≥ 30 breaths/min (*OR* 0.296, *95% CI* 0.136–0.640, *P* = 0.002) were negatively related to hMPV-p. Sputum production (*OR* 13.418, *95% CI* 6.769–26.598, *P* < 0.001) was positively associated with hPIV-p, while nasal congestion (*OR* 0.194, *95% CI* 0.098–0.387, *P* < 0.001), dyspnea (*OR* 0.469, *95% CI* 0.272–0.809, *P* < 0.001), and respiratory rate ≥30 breaths/min (*OR* 0.090, *95% CI* 0.032–0.257, *P* < 0.001) on admission were negatively related to hPIV-p. After adjustment for confounders, multivariate logistic regression analysis suggested that hMPV-p (*OR* 0.355, *95% CI* 0.135–0.932, *P* = 0.035) and hPIV-p (*OR* 0.311, *95% CI* 0.121–0.784, *P* = 0.013) were associated with decreased 30-day mortality compared with RSV-p. RSV infection (*OR* 4.183, *95% CI* 1.709–10.236, *P* = 0.002) was identified as an independent predictor of 30-day mortality in patients with PMV-p.

**Conclusion:** RSV-p caused more severe disease than hMPV-p and hPIV-p. Although some clinical features are helpful for distinguishing the diseases, etiologic diagnosis is critical in the management of the PMV-p.

## Introduction

Despite the current advances in medical technology and the economy, community-acquired pneumonia (CAP) is still a leading cause of death due to infectious diseases globally (1). Respiratory syncytial virus (RSV), human metapneumovirus (hMPV), and human parainfluenza virus (hPIV) are single-stranded, negative-sense, and enveloped RNA viruses belonging to the Paramyxoviridae family (2). Each year, these viruses cause upper and lower respiratory tract infections, leading to considerable morbidity and mortality worldwide (3, 4). RSV is the major pathogen responsible for CAP in childhood, especially in children under 5 years old (5). hMPV and hPIV are also commonly detected in pediatric patients (6). In recent years, due to the widespread application of molecular diagnostic techniques in the clinic, these paramyxoviruses (PMVs) have become increasingly recognized as potential etiologies of CAP in adults (7–9). In the EPIC (Etiology of Pneumonia in the Community) study, a pathogen was detected in 853 cases among 2,259 adult patients hospitalized with CAP in the United States. Among these cases, hMPV, RSV, and hPIV accounted for 10.3, 8.0, and 7.9% of the pathogens, respectively, ranking the fourth to the sixth most common pathogens, following rhinovirus, influenza virus, and *Mycoplasma pneumoniae* (7). The international GLIMP (Global initiative for methicillin-resistant *Staphylococcus aureus* pneumonia) study showed that the frequencies of RSV and hMPV in adults with CAP were 2–6% and 0–3.1%, respectively (8). A meta-analysis including 21 studies from Europe indicated that the pooled proportions of hPIV [3%, *95% confidence interval* (*CI*) 2–5%], RSV (2%, *95% CI* 1–3%), and hMPV (2%, *95% CI* 1–2%) in adult CAP patients were similar, and the detection rates were second only to influenza virus and rhinovirus in viral pneumonia (9). Despite the relatively low incidence of CAP, the overall mortality caused by the PMV-p was comparable to, or even higher than, that of influenza pneumonia, especially in elderly patients and patients with chronic underlying conditions (10, 11).

However, in terms of real-world information, except for RSV-related pneumonia (RSV-p), current knowledge about hMPV-related pneumonia (hMPV-p) and hPIV-related pneumonia (hPIV-p) is scarce. Additionally, previous research on PMV-related pneumonia (PMV-p) related to these three viruses has been focused mainly on specific populations, such as pediatric and immunocompromised patients or in health-care settings (12, 13). Therefore, the generalizability of the conclusions drawn from these studies is limited.

In this study, we conducted a multicenter, real-world study, investigating the clinical features and outcomes of immunocompetent adult patients with the three PMV-p onset in community, with the aims to: (i) explore the clinical possibility of distinguishing between them; (ii) evaluate the impact of specific virus types on the disease severity of pneumonia.

## Materials and Methods

### Study Design and Patient Recruitment

Hospitalized patients who tested positive for RNA of RSV, hMPV, and hPIV in respiratory specimens were screened. Virus detection was performed at the microbiology laboratories of five tertiary hospitals in China between January 1, 2011, and December 31, 2019. Each hospital conducted the testing for viral RNA independently. Details of these participating centers are listed in Supplementary Material 1. Patients with laboratory and radiologically confirmed RSV-p/hMPV-p/hPIV-p were included. The following criteria were applied (14, 15): (i) patients aged under 18 years; (ii) patients who were not diagnosed with community-onset pneumonia [i.e., pneumonia onset ≥48 h post-admission and hospitalized within the previous 28 days (16)]; (iii) patients affected by immunocompromised factors.

### Disease and Treatment Definitions

RSV-p/hMPV-p/hPIV-p patients were defined as those individuals who tested positive result for RSV/hMPV/hPIV RNA by reverse transcription-polymerase chain reaction (RT-PCR) analysis of respiratory specimens (i.e., nasal/nasopharyngeal swabs, sputum, bronchial aspirates, or bronchoalveolar lavage fluid) and also exhibited respiratory symptoms, together with newly emerging pulmonary infiltrates detected by chest radiograph. Systemic corticosteroid usage was defined as the administration of least one dose of any systemic corticosteroid during hospitalization. Community-acquired respiratory conditions with coinfecting pathogens were defined as cases of pathogen identification within the first 48 h after admission using standard microbiologic procedures (17). The criteria for definition of microbiological coinfection are shown in Supplementary Material 2.

### Data Collection

Patient data were retrospectively extracted from electronic records using a standardized case report form. These data included: (i) date of admission and demographic information, (ii) underlying disease (comorbidities are illustrated in Supplementary Material 3), (iii) clinical symptoms, (iv) vital signs, (v) laboratory and radiological findings at admission, (vi) community-acquired respiratory coinfections, and (vii) management and outcomes [i.e., administration of antibiotics and systemic corticosteroids, invasive and noninvasive mechanical ventilation, admittance to the intensive care unit (ICU), and 30-day mortality]. Patients hospitalized for <30 days were followed up by phone to determine their survival status.

### Statistical Analysis

Continuous data were analyzed for normality by the Kolmogorov–Smirnov test. Measurement data with a normal distribution were presented as the mean ± standard deviation (SD). Data with a non-normal distribution were expressed as the median. Categorical variables were analyzed using the chi-square test or Fisher's exact test. Continuous variables were analyzed using Student's *t*-test or Mann–Whitney *U*-test. *P* values ≤ 0.05 were considered to indicate statistical significance. All probability tests were two-tailed.

We compared the demographical and baseline clinical features between patients with hMPV-p/hPIV-p and RSV-p. Variables with *P* < 0.05 in univariate analysis were entered into the multivariate backstep logistic regression to identify the predictors of hMPV-p and hPIV-p.

To evaluate the association between etiologic categories (RSV-p, reference) with the clinical outcomes (invasive/noninvasive ventilation, ICU admission, and 30-day mortality), we performed multivariate logistic regression after adjustment for age, sex, duration of illness from onset to admission, comorbidities, obesity, smoking history, systemic corticosteroid use, and coinfection with other pathogens. These known risk factors previously reported to be associated with the clinical outcomes of patients with respiratory virus infections served as confounders.

According to the survival status at 30 days post-admission, the full sample of the three PMV-p patients was divided into deceased and survival groups. The baseline characteristics of the two groups were then compared. To explore the risk factors for 30-day mortality, variables with *P* < 0.05 in the univariate analysis were entered into the multivariate logistic regression analysis. All analyses were performed using SPSS version 22.0.

## Results

### Screening Process

**Figure 1:**
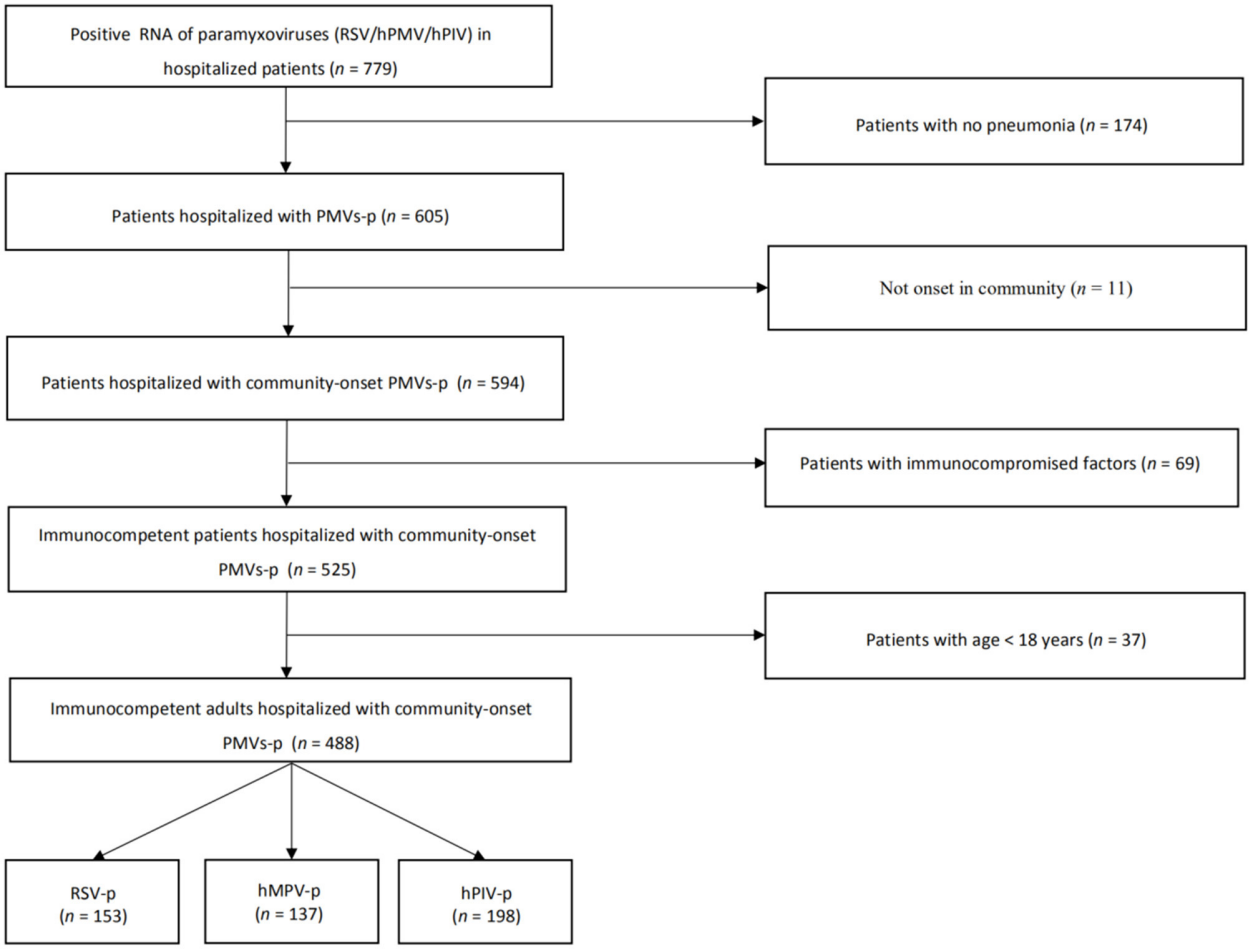
Screening algorithm of patients hospitalized with the three types of PMVs-p.

We screened 779 patients who tested positive for RSV, hMPV, or hPIV RNA. A total of 153 RSV-p patients, 137 hMPV-p, and 198 hPIV-p patients were included in the study; all patients were laboratory and radiologically confirmed (Figure 1).

### Seasonal Distribution of the Three Groups of Paramyxovirus-Related Pneumonia Patients

The seasonal distribution of the three groups of PMV-p patients in our study is shown in Supplementary Figure 1. In general, the cases of RSV-p and hMPV-p showed similar seasonality, with distribution from November to May and the peak during January to March. The prevalence of hPIV-p increased from March to September, with the peak during May to June.

### Overview of the Total Paramyxovirus-Related Pneumonia Patients

Of the 488 PMV-p patients, the median age was 65.0 years (interquartile range, 54.0–71.0 years). Males accounted for 55.5% (271/488). The top 3 underlying diseases were cardiovascular disease (33.8%, 165/488), chronic obstructive pulmonary disease (COPD) (21.1%, 103/488), and cerebrovascular disease (16.0%, 78/488). Cough (94.7%, 462/488), fever ≥38°C (67.8%, 331/488), and dyspnea (59.6%, 291/488) were the most common symptoms at admission. Among the patients, 28.7% (140/488) had pO_2_/FiO_2_ in <250 mmHg. Multilobar infiltrates and pleural effusion in chest radiology were seen in 82.2% (401/488) and 22.3% (109/488) of patients, respectively (Table 1).

**Table 1:** Demographic and clinical features of patients with the three types of PMVs-p.

**Variable**	**Total** **(*n* = 488)**	**RSV-p** **(*n* = 153)**	**hMPV-p** **(*n* = 137)**	***p_**1**_* value**	**hPIV-p** **(*n* = 198)**	***p_**2**_* value**
Age (years, median, IQR)	65.0 (54.0–71.0)	69.0 (60.0–72.0)	69.0 (60.5–74.0)	0.674	54.0 (38.0–67.3)	**<0.001**
Age ≥ 65 years (*n*, %)	247 (50.6)	94 (61.4)	75 (54.7)	0.248	78 (39.4)^#^	**<0.001**
Male (*n*, %)	271 (55.5)	88 (57.5)	85 (62.0)	0.433	98 (49.5)	0.135
Days from illness onset to admission (median, IQR)	3.0 (2.0–5.0)	4.0 (3.0–6.0)	3.5 (2.0–4.5)	**<0.001**	3.0 (2.0–5.0)	**<0.001**
**Comorbidities (*****n*****, %)**						
Cardiovascular disease	165 (33.8)	55 (35.9)	48 (35.0)	0.871	62 (31.3)	0.361
COPD	103 (21.1)	39 (25.5)	40 (29.2)	0.479	24 (12.1)^#^	**0.001**
Cerebrovascular disease	78 (16.0)	23 (15.0)	24 (17.5)	0.566	31 (15.7)	0.872
Diabetes mellitus	55 (11.3)	26 (17.0)	14 (10.2)	0.095	15 (7.6)^#^	**0.006**
Chronic kidney disease	40 (8.2)	20 (13.1)	5 (3.6)^#^	**0.004**	15 (7.6)	0.088
Solid malignant tumor	35 (7.2)	20 (13.1)	7 (5.1)^#^	**0.020**	8 (4.0)^#^	**0.002**
Asthma	18 (3.7)	6 (3.9)	8 (5.8)	0.447	4 (2.0)	0.460
Obesity (*n*, %)	48 (9.8)	10 (6.5)	15 (10.9)	0.181	23 (11.6)	0.106
Smoking history (*n*, %)	141 (28.9)	44 (28.8)	40 (29.2)	0.934	57 (28.8)	0.995
**Baseline clinical and radiologic features (*****n*****, %)**						
Fever ≥ 38°C	331 (67.8)	108 (70.6)	90 (65.7)	0.371	133 (67.2)	0.494
Nosal congestion	81 (16.6)	50 (32.7)	7 (5.1) ^#^	**<0.001**	24 (12.1)^#^	**<0.001**
Rhinorrhea	111 (22.7)	44 (28.8)	25 (18.2)^#^	**0.036**	42 (21.2)^#^	0.103
Sore throat	73 (15.0)	27 (17.6)	23 (16.8)	0.847	23 (11.6)	0.109
Myalgia	69 (14.1)	26 (17.0)	19 (13.9)	0.463	24 (12.1)	0.195
Cough	462 (94.7)	147 (96.1)	131 (95.6)	0.854	184 (92.9)	0.207
Sputum production	176 (36.1)	17 (11.1)	46 (33.6)^#^	**<0.001**	113 (57.1)^#^	**<0.001**
Chest pain	102 (20.9)	31 (20.3)	37 (27.0)	0.176	34 (17.2)	0.460
Dyspnea	291 (59.6)	109 (71.2)	100 (73.0)	0.740	82 (41.4)^#^	**<0.001**
Mental confusion	79 (16.2)	20 (13.1)	29 (21.2)	0.066	30 (15.2)	0.580
Respiratory rates ≥ 30 breaths/min	55 (11.3)	34 (22.2)	14 (10.2)	**0.006**	7 (3.5)^#^	**<0.001**
SBP <90 mmHg	4 (0.8)	2 (1.3)	2 (1.5)	1.000	0 (0.0)	0.189
Leukocytes > 10 × 10^9^/L	118 (24.2)	38 (24.8)	25 (18.2)	0.174	55 (27.8)	0.536
Lymphocytes < 0.8 × 10^9^/L	64 (13.1)	23 (15.0)	22 (16.1)	0.810	19 (9.6)	0.120
HB <100 g/L	94 (19.3)	37 (24.2)	18 (13.1)^#^	**0.017**	39 (19.7)	0.312
ALB <30 g/L	31 (6.4)	8 (5.2)	11 (8.0)	0.321	12 (6.1)	0.739
BUN > 7 mmol/L	179 (36.7)	62 (40.5)	71 (51.8)	0.054	46 (23.2)^#^	**0.001**
PO_2_/FiO_2_ <250 mmHg	140 (28.7)	42 (27.5)	37 (27.0)	0.932	61 (30.8)	0.493
Multilobar infiltrates	401 (82.2)	117 (76.5)	111 (81.0)	0.400	173 (87.4)	0.264
Pleural effusion	109 (22.3)	34 (22.2)	35 (25.5)	0.507	40 (20.2)	0.645
Coinfection (*n*, %)	149 (30.5)	53 (34.6)	35 (25.5)	0.093	61 (30.8)	0.447

*PMVs-p, paramyxoviruses-related pneumonia; RSV-p, respiratory cyncytial virus-related pneumonia; hMPV-p, human metapneumovirus-related pneumonia; hPIV-p, human parainfluenza virus-related pneumonia; IQR, interquartile range; COPD, chronic obstructive pulmonary disease; SBP, systolic blood pressure; HB, hemoglobin; ALB, albumin; BUN, blood urea nitrogen; PO_2_/FiO_2_, arterial pressure of oxygen/fraction of inspiration oxygen. ^#^: variables cited in the table above were the candidates which were entered into the multivariate logistic regression model. p_1_: hMPV-p vs. RSV-p, p_2_: hPIV-p vs. RSV-p. The bolded values are p-values <0.05, which represented significant differences between patients with hMPV-p/hPIV-p and RSV-p*.

An additional community-acquired coinfected pathogen was isolated from 30.5% (149/488) of patients. The most common etiology was *Klebsiella pneumoniae* (43.0%, 64/149), followed by *Streptococcus pneumoniae* (15.4%, 23/149) and *Staphylococcus aureus* (14.1%, 21/149) (Supplementary Material 4).

All patients were treated with antibiotics after admission, and 9.4% (46/488) received systemic corticosteroids. Noninvasive ventilation and invasive ventilation were conducted in 8.0% (39/488) and 9.6% (47/488) of patients, respectively. During hospitalization, 12.3% (60/488) of patients developed respiratory failure. The complications of heart failure and septic shock were observed in 10.9% (53/488) and 3.5% (17/488) of patients, respectively. A total of 11.3% (55/488) of patients were admitted to the ICU, and the all-cause 30-day mortality was 8.8% (43/488) (Table 2).

**Table 2:** Clinical management and outcomes of patients with three types of PMVs-p.

**Variable**	**Total** **(*n* = 488)**	**RSV-p** **(*n* = 153)**	**hMPV-p** **(*n* = 137)**	***p*_**1**_ value**	**hPIV-p** **(*n* = 198)**	***p_**2**_*value**
Systemic corticosteroids use (*n*, %)	46 (9.4)	15 (9.8)	16 (11.7)	0.606	15 (7.6)	0.448
Noninvasive ventilation (*n*, %)	39 (8.0)	17 (11.1)	14 (10.2)	0.806	8 (4.0)	**0.011**
Invasive ventilation (*n*, %)	47 (9.6)	20 (13.1)	12 (8.8)	0.242	15 (7.6)	0.088
Vasopressor use (*n*, %)	17 (3.5)	11 (7.2)	3 (2.2)	0.047	3 (1.5)	**0.007**
**Complications (*****n*****, %)**						
Respiratory failure	60 (12.3)	39 (25.5)	10 (7.3)	**0.001**	11 (5.6)	**<0.001**
Heart failure	53 (10.9)	31 (20.3)	12 (8.8)	**0.006**	10 (5.1)	**<0.001**
Septic shock	17 (3.5)	12 (7.8)	3 (2.2)	**0.030**	2 (1.0)	**0.001**
Acute renal failure	10 (2.0)	6 (3.9)	4 (2.9)	0.885	0 (0.0)	**<0.001**
Admittance to ICU (*n*, %)	55 (11.3)	30 (19.6)	13 (9.5)	**0.015**	12 (6.1)	**<0.001**
Length of stay in hospital (days, median, IQR)	10.0 (8.0–14.0)	14.0 (10.0–19.0)	11.0 (10.0–17.0)	**<0.001**	8.0 (4.0–9.0)	**<0.001**
30-day mortality (*n*, %)	43 (8.8)	22 (14.4)	9 (6.6)	**0.032**	12 (6.1)	**0.009**

*ICU, intensive care unit. p_1_, hMPV-p vs. RSV-p, p_2_, hPIV-p vs. RSV-p. The bolded values are p-values < 0.05, which represented significant differences between patients with hMPV-p/hPIV-p and RSV-p*.

### Factors Associated With Human Metapneumovirus-Related Pneumonia and Human Parainfluenza Virus-Related Pneumonia Patients

**Figure 2:**
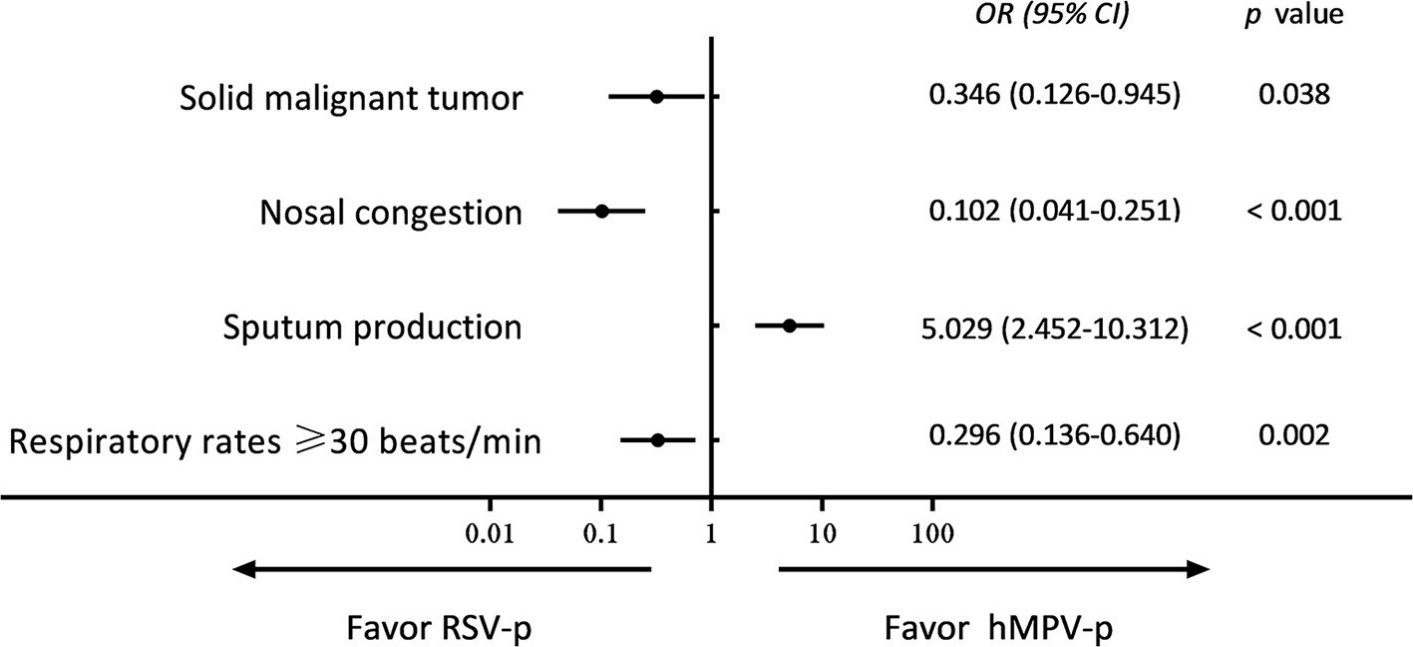
Multivariate analysis of predictors for hMPV-p (compared with RSV-p).

Compared with RSV-p patients, a higher proportion of hMPV-p patients showed sputum production (33.6 vs. 11.1%, *P* < 0.001), whereas the proportions of patients with chronic kidney disease (3.6 vs. 13.1%, *P* = 0.004), solid malignant tumor (5.1 vs. 13.1%, *P* = 0.020), nasal congestion (5.1 vs. 32.7%, *P* < 0.001), rhinorrhea (18.2 vs. 28.8%, *P* = 0.036), respiratory rates ≥ 30 breaths/min (10.2 vs. 22.2%, *P* = 0.006), and hemoglobin <100 g/L (13.1 vs. 24.2%, *P* = 0.017) were all lower (Table 1). A multivariate logistic regression model revealed that sputum production [*odds ratio* (*OR*) 5.029, *95% CI* 2.452–10.312, *P* < 0.001] was positively associated with hMPV-p, whereas solid malignant tumor (*OR* 0.346, *95% CI* 0.126–0.945, *P* = 0.038), nasal congestion (*OR* 0.102, *95% CI* 0.041–0.251, *P* < 0.001), and respiratory rates ≥ 30 breaths/min (*OR* 0.296, *95% CI* 0.136–0.640, *P* = 0.002) were negatively related to hMPV-p (Figure 2).

**Figure 3:**
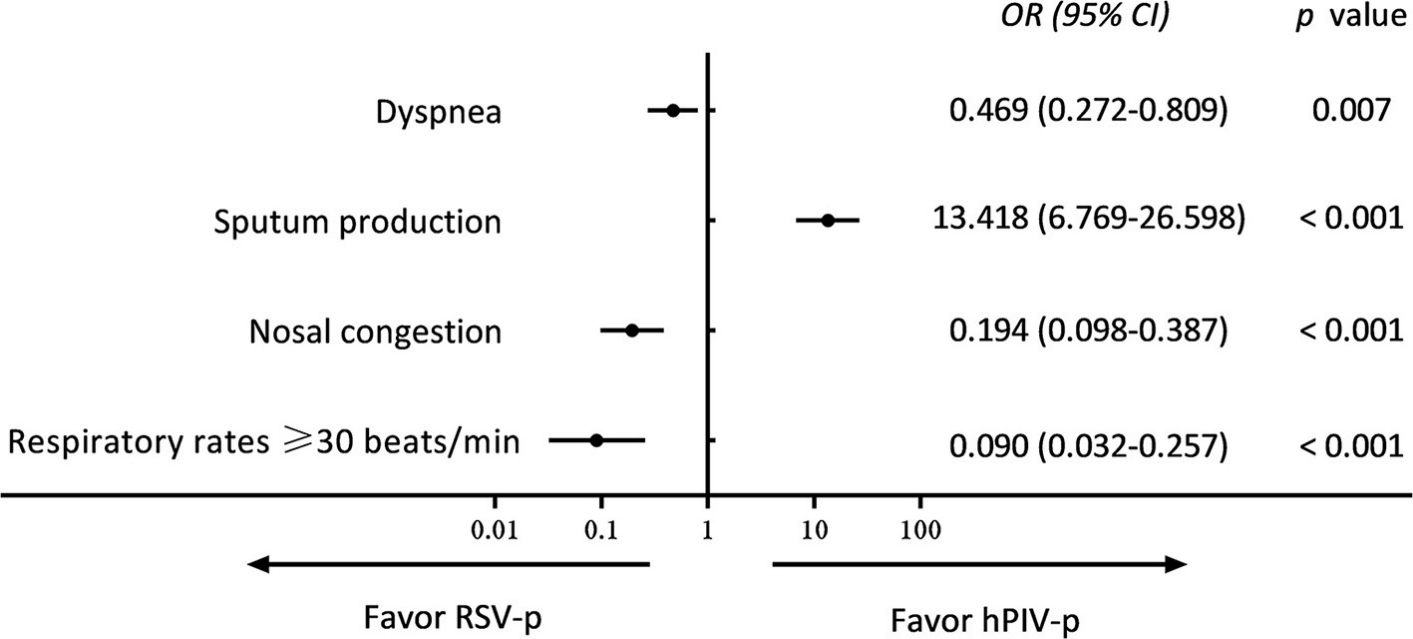
Multivariate analysis of predictors for hPIV-p (compared with RSV-p).

Compared with RSV-p patients, the hPIV-p patients were younger (54.0 vs. 69.0 years, *P* < 0.001), and a higher proportion exhibited sputum production (57.1 vs. 11.1%, *P* < 0.001), while the proportions of hPIV-p patients aged ≥65 years (39.4 vs. 61.4%, *P* < 0.001), with diabetes mellitus (7.6 vs. 17.0%, *P* = 0.006), COPD (12.1 vs. 25.5%, *P* = 0.001), solid malignant tumor (4.0 vs. 13.1%, *P* = 0.002), nasal congestion (12.2 vs. 32.7%, *P* < 0.001), dyspnea (41.4 vs. 71.2%, *P* < 0.001), respiratory rates ≥ 30 breaths/min (3.5 vs. 22.2%, *P* < 0.001), and blood–urea–nitrogen (BUN) > 7 mmol/L (23.2 vs. 40.5%, *P* = 0.001) were all lower (Table 1). A multivariate logistic regression model indicated that sputum production (*OR* 13.418, *95% CI* 6.769–26.598, *P* < 0.001) was positively associated with hPIV-p, whereas nasal congestion (*OR* 0.194, *95% CI* 0.098–0.387, *P* < 0.001), dyspnea (*OR* 0.469, *95% CI* 0.272–0.809, *P* < 0.001), and respiratory rates ≥ 30 breaths/min (*OR* 0.090, *95% CI* 0.032–0.257, *P* < 0.001) were negatively related to hPIV-p (Figure 3).

### Impact of Virus Types on Clinical Outcomes

In the univariate analysis, compared with RSV-p, hMPV-p was associated with decreased likelihood of ICU admission (*OR* 0.430, *95% CI* 0.214–0.863, *P* = 0.018) and 30-day mortality (*OR* 0.419, *95% CI* 0.186–0.944, *P* = 0.036) but not with invasive ventilation (*OR* 0.603, *95% CI* 0.285–1.278, *P* = 0.187). The same associations were also seen for hPIV-p with ICU admission (*OR* 0.336, *95% CI* 0.174–0.651, *P* = 0.001), 30-day mortality (*OR* 0.384, *95% CI* 0.184–0.804, *P* = 0.011), and invasive ventilation (*OR* 0.515, *95% CI* 0.256–1.037, *P* = 0.063) (Table 3).

**Table 3:** The impact of virus types on the clinical outcomes of PMVs-p.

**Clinical outcomes**	**Virus type** **(ref: RSV)**	**Univariate logistic analysis**		**Multivariate logistic analysis**	
		***OR (95% CI)***	***p* value**	*a*OR (95% CI)*	***p* value**
Invasive ventilation	hMPV	0.603 (0.285–1.278)	0.187	0.162 (0.057–0.459)	0.001
	hPIV	0.515 (0.256–1.037)	0.063	0.304 (0.107–0.862)	0.025
Admittance to ICU	hMPV	0.430 (0.214–0.863)	0.018	0.248 (0.105–0.586)	0.001
	hPIV	0.336 (0.174–0.651)	0.001	0.385 (0.164–0.904)	0.028
30-day mortality	hMPV	0.419 (0.186–0.944)	0.036	0.355 (0.135–0.932)	0.035
	hPIV	0.384 (0.184–0.804)	0.011	0.311 (0.121–0.784)	0.013

OR, odd ratio; CI, interval confidence. *: adjusted for age, sex, days from illness onset to admisssion, comorbidities (cardiovascular disease, cerebrovascular disease, diabete mellitus, chronic pulmonary disease, asthma, chronic kidney disease and solid malignant tumor), obesity, smoking history, coinfection and systemic corticosteroids usage.

**Figure 4:**
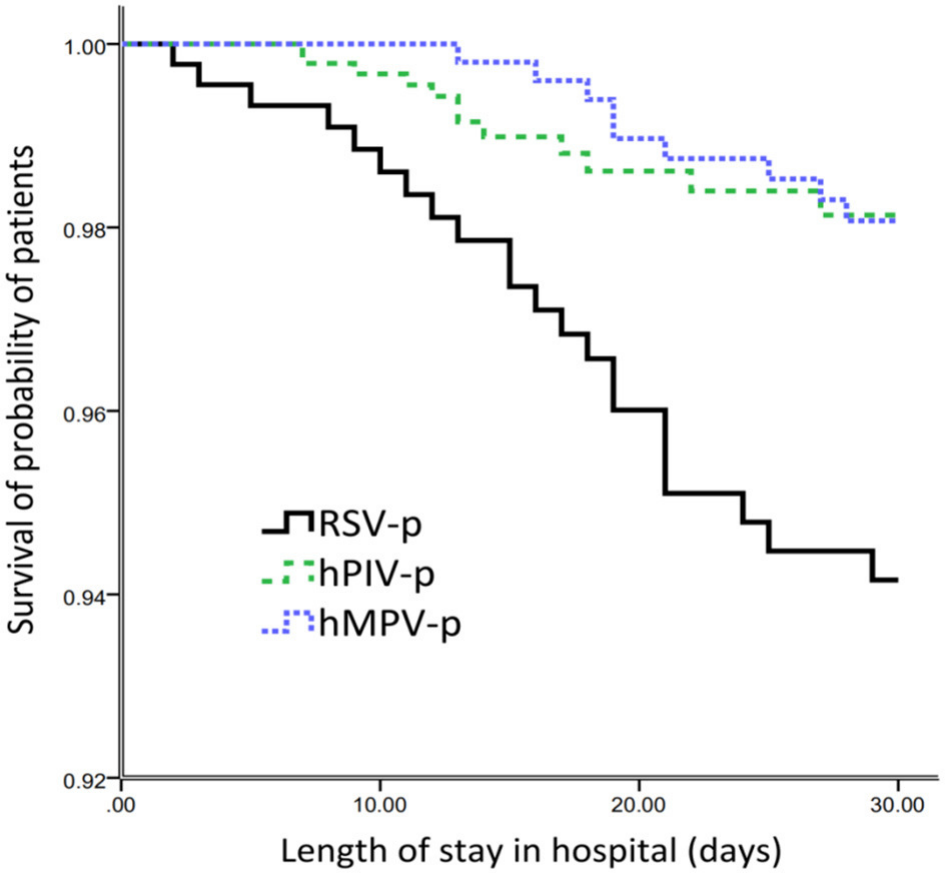
Survival rate of patients hospitalized with the three types of PMVs-p (censored at 30d after admission).

After adjusting for age, sex, days from the onset of illness to hospital admission, comorbidities (cardiovascular disease, cerebrovascular disease, diabetes mellitus, COPD, asthma, chronic kidney disease, and solid malignant tumor), obesity, smoking history, coinfection, and systemic corticosteroid usage, multivariate analysis revealed that, compared with RSV-p, hMPV-p was associated with decreased likelihood of invasive ventilation (*OR* 0.162, *95% CI* 0.057–0.459, *P* = 0.001), ICU admission (*OR* 0.248, *95% CI* 0.105–0.586, *P* = 0.001), and 30-day mortality (*OR* 0.355, *95% CI* 0.135–0.932, *P* = 0.035). The ORs of hPIV-p for invasive ventilation, ICU admission, and 30-day mortality were 0.304 (*95% CI* 0.107–0.862, *P* = 0.025), 0.385 (*95% CI* 0.164–0.904, *P* = 0.028), and 0.311 (*95% CI* 0.121–0.784, *P* = 0.013), respectively (Table 3) and Figure 4.

### Risk Factors for 30-Day Mortality in the Three Types of Paramyxovirus-Related Pneumonia Patients

To explore the risk factors for 30-day mortality among all the PMV-p patients, the following variables were entered into the backstep logistic regression model: age ≥ 65 years, RSV infection, diabetes mellitus, COPD, solid malignant tumor, smoking history, mental confusion, lymphocytes <0.8 × 10^9^/L, hemoglobin <100 g/L, serum albumin <30 g/L, BUN > 7 mmol/L, PO_2_/FiO_2_ <250 mmHg, and systemic corticosteroid use (Supplementary Material 5). And age ≥ 65 years (*OR* 6.766, *95% CI* 2.009–22.785, *P* = 0.002), RSV infection (*OR* 4.183, *95% CI* 1.709–10.236, *P* = 0.002), smoking history (*OR* 4.162, *95% CI* 1.659–11.442, *P* = 0.002), lymphocytes <0.8 × 10^9^/L (*OR* 6.786, *95% CI* 2.873–16.032, *P* < 0.001), serum albumin <30 g/L (*OR* 8.495, *95% CI* 1.584–45.547, *P* < 0.001), and systemic corticosteroid use (*OR* 12.229, *95% CI* 4.174–35.831, *P* < 0.001) were confirmed to be independently associated with 30-day mortality of PMV-p patients (Table 4).

**Table 4:** Risk factors for 30-day mortality of three types of PMVs-p.

**Variable**	***OR (95% CI)***	***p* value**
Age ≥ 65 years	6.766 (2.009–22.785)	0.002
RSV infection	4.183 (1.709–10.236)	0.002
Smoking history	4.162 (1.659–11.442)	0.002
ALB < 30 g/L	8.495 (1.584–45.547)	0.013
Lymphocytes <0.8 × 10^9^/L	6.786 (2.873–16.032)	<0.001
Systemic corticosteroids use	12.229 (4.174–35.831)	<0.001

## Discussion

Previous studies have indicated that different respiratory viruses show unique seasonal patterns (18, 19). A multicenter, population-based, prospective surveillance study of nine respiratory viruses among CAP patients in China suggested that RSV and hMPV presented the same seasonality with peaks during the period from winter to spring, while hPIV peaked slightly later in the summer (19). A similar pattern was also observed in our study, despite the retrospective data collection. However, the seasonal epidemiology might show some differences between temperate and tropical/subtropical climates (20).

Generally, in our study, the clinical and radiologic characteristics of patients with the three types of PMV-p were similar. Cough, fever, and dyspnea were the most common symptoms. However, by detailed comparison, we identified certain clinical features that could actually be used for differential diagnosis. Solid malignant tumor, nasal congestion, dyspnea, and respiratory rates ≥ 30 beats/min were more likely to be associated with RSV-p, while sputum production was more often related to hMPV-p and hPIV-p. These findings are consistent with previous research. For example, a multicenter, retrospective study in France showed that, in comparison with RSV+ patients, hMPV+ patients were less likely to have cancer (*OR* 0.4, *95% CI* 0.2–0.9) (10). By comparing patients hospitalized with RSV and influenza, Lee et al. (21) found that patients with RSV more often had dry cough, wheezy breathing, and dyspnea. Walsh et al. (22) showed that, compared with other viral infections in adults aged ≥65 years, RSV infection was associated with symptoms of nasal congestion (*OR* 2.0, *95% CI* 1.3–2.9) and wheezing (*OR* 1.8, *95% CI* 1.2–2.8). Howard et al. (23) compared the clinical features of adults with hMPV-p with those of adults with RSV-p using population-based surveillance data from eight hospitals in the US and found that dyspnea and wheezing were more common in RSV-p patients. Wheezing seems to be a characteristic of RSV infection among infants (24), as well as in adults (21–23). It can be speculated that this association is due to the specific tropism of RSV for small airway epithelial cells and the common involvement of the lower airways. Resultant edema and inflammatory changes cause airflow obstruction (25). In addition, nasal or lower airway replication of RSV may induce a dominant Th2-type cellular immune response, with RSV-specific IgE production and leukotriene secretion, resulting in bronchospasm (26). The increase in allergic sensitization of the airways persists even after the RSV infection is cleared (27). Given the limited population, some features (e.g., solid tumor), although statistically significant, should probably not be suggested as diagnostic features of RSV pneumonia in adults. In addition, the absolute values of the differences in these clinical indicators between PMV-p patients were only 10–20%, indicating that these are helpful for differential diagnosis, particularly in primary and resource-limited hospitals, but should not be used as substitutes for etiological tests.

Previous studies showed that the mortality of PMV infection among adults is 0–50%, depending on the virus type, immune status of the host, and severity of disease (28–30), which is consistent with our results. However, most of these studies focused on lower respiratory tract infections or acute respiratory infections affected by these PMVs. To the best of our knowledge, only one study compared the outcomes of hMPV-p with pneumonia caused by other viruses. In this research, the severe outcomes (including in-hospital death, acute respiratory distress syndrome, need for extracorporeal membrane oxygenation, and/or requiring mechanical ventilation) occurred in 6 and 10% of adult patients hospitalized with hMPV-p and RSV-p, respectively (23). After controlling for confounders (such as age, sex, ethnicity, presence of a comorbidity, antibiotics prior to admission, and vaccination against influenza and pneumococcal infections), RSV (*OR* 1.82, *95% CI* 1.32–2.50) was found to cause more severe disease than hMPV, while all other viruses showed a similar disease severity in adult patients with CAP. Again, this conclusion was proven by our findings. Furthermore, the outcomes of infectious diseases were influenced by hosts, pathogens, and environments (31). In some studies, clinical outcomes were only compared directly with disease severity of the three PMV infections and were not controlled for confounders (10, 21, 23, 28, 29, 32). In our study, after adjustment for numerous potential confounders, we found that, compared with hMPV-P and hPIV-p, RSV-p was associated with increased risks for invasive ventilation, ICU admission, and 30-day mortality. Our findings help to clarify the direct influence of virus types on clinical outcomes. To further minimize the bias, we used another methodology to control for the potential confounders. The results obtained using both methodologies confirmed the association of RSV-p with increased mortality, which enhanced the reliability of our conclusions.

Some limitations of our current study should be pointed out. First, despite our representative sample size and comprehensive statistical analysis approach, the retrospective design of this study is prone to some unavoidable selection biases. For instance, nucleic acid tests were performed based on the subjective judgment of the attending physicians. Thus, it is possible that more severe (or milder) patients were more (or less) likely to be tested and, as a result, not all cases of respiratory disease were eligible for swabbing, thus leading to a form of selection. However, our study presents the real-world profile of the three types of PMV-p. Second, other respiratory tract viruses were not routinely detected; thus, we could not exclude coinfection with other viruses (6, 33). Third, some studies suggested differences in the clinical features and outcomes among the subtypes of PMV infections. Further work is required to compare the clinical features according to virus subtypes. Finally, our study population consisted of immunocompetent adult hospitalized patients. Thus, our conclusions should be assessed cautiously before applying them to immunocompromised patients, pediatric patients, and outpatients.

In conclusion, our study shows that the disease caused by RSV-p is more severe than that caused by hMPV-p and hPIV-p. Although some clinical features are helpful for discriminating between the pneumonias caused by the three PMVs, the differences in the outcomes highlight the importance of virus strain testing in the clinical management of PMV-p. Additionally, our results provide a theoretical basis for the priority given to the development of antiviral medications and vaccines in public health.

## Data Availability Statement

The original contributions presented in the study are included in the article/Supplementary Materials, further inquiries can be directed to the corresponding author/s.

## Ethics Statement

The studies involving human participants were reviewed and approved by Ethics Committee of Beijing Jishuitan Hospital (No.201911-15). Written informed consent for participation was not required for this study in accordance with the national legislation and the institutional requirements.

## Author Contributions

LC and XH contributed to the study concept and design. LC, XH, YL, CZ, and XX contributed to the acquisition of data. LC contributed to the statistical analysis of the data and drafting of the manuscript. XH and XX contributed to critical revision of the manuscript for important intellectual content. All authors agree with the article submission, read and approved the final manuscript.

## Conflict of Interest

The authors declare that the research was conducted in the absence of any commercial or financial relationships that could be construed as a potential conflict of interest.
