# Supplementary material for: The Clinical Characteristics and Outcomes of Adult Patients With Pneumonia Related to Three Paramyxoviruses
Source: Front Med (Lausanne). 2021 Jan 18;7:574128. doi: 10.3389/fmed.2020.574128 (PMC7848145; doi:10.3389/fmed.2020.574128)
Supplement: Supplementary file 1 [file Data_Sheet_1.DOC]

**Supplementary material 1 Details of participating centers**

| **Name of the hospital** | **Province, city** | **Teaching Hospital** | **Beds** | **Staffs of Clinical Microbioloy Lab** |
| --- | --- | --- | --- | --- |
| Beijing Jishuitan Hospital | Beijing | Yes | 1500 | 10 |
| Beijing Chao-Yang Hospital | Beijing | Yes | 1400 | 11 |
| **the 2nd People’s Hospital of Yunnan Province** | **Kunming,**  **Yan’an** | Yes | 1302 | 4 |
| **Qingdao Municipal Hospital** | ShanDong,  Qingdao | Yes | 1200 | 4 |
| Beijing Huimin Hospital | Beijing | Yes | 1000 | 2 |

**Supplementary material 2 Definition of microbiological criteria of coinfected with other pathogens**

if one of the following criteria was met:

1. Positive urinary antigen for *Legionella pneumophila* ;
2. Positive urinary antigen for *Streptococcus pneumoniae* ;
3. Positive bacterial culture from blood or plural fluid except for coagulase negative *Staphylococcus spp*.
4. Paired sera with a fourfold or more increase in the titers of antibodies to *Mycoplasma pneumoniae* (MP), *Chlamydia pneumonia*, *L pneumophila or* respiratory viruses (Parainfluenza, Adenovirus, Respiratory syncytial virus)*.* Or Serum IgM antibody (MIF) ≥ 1:16 for *Chlamydia pneumonia.*
5. Detection of respiratory virus in sputum/bronchoalveolar lavage (BALF)/throat swabs by Realtime-PCR according to manufacturer’s instructions, including respiratory syncytial virus (RSV) types A and B, parainfluenza virus (PIV) types 1, 2, 3 and 4, rhinovirus (HRV), enterovirus (EV), coronavirus (hCoV) types 229E, NL63, OC43 and HKU1, parapneumovirus (hMPV), and adenovirus (AdV), bocavirus;
6. Bacteria isolated form purulent sputum (defined as an adequate quality sputum sample with > 25 leukocytes and < 10 epithelial cells per × 100 magnification field) with compatible findings of Gram staining;
7. Detection of *Mycoplasma pneumoniae* (MP), *Chlamydia pneumonia* or *L pneumophila* in sputum/BALF/throat swabs by Realtime-PCR;
8. Serum IgM antibody positive for *Mycoplasma pneumoniae* (MP), or Serum IgG antibody (MIF) ≥ 1:512 for *Chlamydia pneumonia.*
9. Invasive pulmonary aspergillosis were diagnosed in accordance with the revised definitions of invasive fungal diseases from the European Organization for Research and Treatment of Cancer and the Mycoses Study Group Education and Research Consortium [1].

References

1. Donnelly JP, Chen SC, Kauffman CA, et al. Revision and Update of the Consensus Definitions of Invasive Fungal Disease From the European Organization for Research and Treatment of Cancer and the Mycoses Study Group Education and Research Consortium. Clin Infect Dis. 2019, pii: ciz1008.

**Supplementary material 3 Definition of underlying diseases**

1. Smoking was defined as cigarette smokers of ten cigarettes/d during at least the previous year；
2. Obesity was defined as body mass index (BMI) ≥ 30 kg/m^2^;
3. Cardiovascular disease included coronary heart disease and chronic congestive heart failure;
4. Coronary heart disease included angina pectoris, myocardial infarction, ischemic cardiomyopathy;
5. Chronic congestive heart failure was defined as cardiomegaly and ejection fraction ≤40%;
6. Cerebrovascular diseases included transient ischemic attack, cerebral hemorrhage, subarachnoid hemorrhage, cerebral infarction, etc;
7. Diebetes mellitus: included diabetes mellitus type 1 and diabetes mellitus type 2, not included impaired glucose tolerance and impaired fasting glycaemia;
8. Chronic obstructive pulmonary disease was defined as: persistent airflow limitation, FEV_1_ / FVC < 70% post bronchodilator;
9. Asthma was defined by the history of respiratory symptoms such as wheeze, cough that varied over time and intensity, together with variable respiratory airway limitation;
10. Chronic kidney disease included diabetic nephropathy, hypertensive renal damage, chronic glomerulonephritis, chronic pyelonephritis, lupus nephritis, IgA nephropathy, nephrotic syndrome, hereditary kidney disease, etc;
11. Immunosuppressive therapy: was defined as systmatic glucocorticosteroid (such as prednisone ≥10mg/d for more than 3 weeks in the last month); cyclosporine or azathioprine use within 3 months, and methotrexate use ≥12.5mg/week within 3 months; biological modifiers such as etanercept and infiximab within 3 weeks.
12. Immunocompromised status included HIV(+)，chemotherapy/radiotherapy within 6 months, immunosuppressive therapy, organ/bone marrow transplantation, splenectomy, hematological neoplasms.
13. Respiratory failure defined as oxygen saturation < 90% on room air or a partial pressure of oxygen from an arterial blood gas < 60 mm Hg on room air;
14. Heart failure was defined as cardiac enlargement and the ejection fraction less than 40%;
15. Septic shock was defined as hypotension requiring use of vasopressors to maintain mean blood pressure of 65 mmHg or greater and having a serum lactate level greater than 2 mmol/L persisting after adequate fluid resuscitation;
16. Acute renal failure was defined as a more than threefold rise in serum creatinine concentration compared to baselinewith an increase of at least 4 mg/dL, accompanied by reduction of urine excretion to <0.3 mL/kg for≥ 24 h or anuria for ≥ 12 h.

**Supplementary material 4 Coinfection with other community-acquired pathogens in patients hospitalized with the three types of PMVs-p**

| **Variable** | **Total**  **(*n* = 488)** | **RSV-p**  **(*n* = 153)** | **hMPV-p**  **(*n* = 137)** | **hPIV-p**  **(*n* = 198)** |
| --- | --- | --- | --- | --- |
| Coinfection (*n*,%) | 149 (30.5) | 53 (34.6) | 35 (25.5) | 61 (30.8) |
| Pathogens (*n*,%) |  |  |  |  |
| *Klebsiella pneumoniae* | 64 (43.0) | 29 (54.7) | 15 (42.9) | 20 (32.8) |
| *Streptococcus pneumoniae* | 23 (15.4) | 4 (7.5) | 4 (11.4) | 15 (24.6) |
| *Staphylococcus aureus* | 21 (14.1) | 10 (18.9) | 5 (14.3) | 6 (9.8) |
| *Other streptococcus spp.* | 13 (8.7) | 3 (5.7) | 5 (14.3) | 5 (8.2) |
| *Haemophilus influenzae* | 7 (4.7) | 0 (0.0) | 1 (2.9) | 6 (9.8) |
| *Moraxelle catarrhalis* | 5 (3.4) | 0 (0.0) | 0 (0.0) | 5 (8.2) |
| *Acinetobacter* | 4 (2.7) | 2 (3.8) | 1 (2.9) | 1 (1.6) |
| *Proteus spp.* | 4 (2.7) | 3 (5.7) | 0 (0.0) | 1 (1.6) |
| *Escherichia coli* | 4 (2.7) | 2 (3.8) | 1 (2.9) | 1 (1.6) |
| *Stenotrophomonas maltophilia* | 2 (1.3) | 1 (1.9) | 0 (0.0) | 1 (1.6) |
| *Pseudomonas aeruginosa* | 2 (1.3) | 1 (1.9) | 1 (2.9) | 0 (0.0) |
| *Chryseobacterium Indole* | 2 (1.3) | 2 (3.8) | 0 (0.0) | 0 (0.0) |
| *Chlalmydia pneumoniae* | 2 (1.3) | 0 (0.0) | 1 (2.9) | 1 (1.6) |
| *Klebsiella acidogens* | 1 (0.7) | 0 (0.0) | 1 (2.9) | 0 (0.0) |
| *Flavobacterium* | 1 (0.7) | 0 (0.0) | 1 (2.9) | 0 (0.0) |
| *Citrobacter spp.* | 1 (0.7) | 0 (0.0) | 1 (2.9) | 0 (0.0) |
| *Mycoplasma pneumoniae* | 1 (0.7) | 0 (0.0) | 0 (0.0) | 1 (1.6) |

4 RSV-p patients, 2 hMPV-p patients and 2 hPIV-p patients were coinfected with ≥ 2 pathogens.

**Supplementary material 5 Comparison of demographic and clinical charateristics between deceased and survival patients with the three types of PMVs-p**

| **Variable** | **Deceased patients**  **(n=43)** | **Survival patients**  **(n=445)** | ***p* value** |
| --- | --- | --- | --- |
| Age ≥ 65 years (*n*, %) ^#^ | 38 (88.4) | 209 (47.0) | **< 0.001** |
| Male (*n*, %) | 26 (60.5) | 245 (55.1) | 0.495 |
| RSV infection (*n*, %) ^#^ | 22 (51.2) | 131 (29.4) | **0.003** |
| Days from illness onset to admission (median, IQR) | 3.0 (2.0-4.0) | 3.0 (2.0-5.0) | 0.345 |
| Comorbidities (*n*, %) |  |  |  |
| Cardiovascular disease | 16 (37.2) | 149 (33.5) | 0.622 |
| Cerebrovascular disease | 11 (25.6) | 67 (15.1) | 0.072 |
| Diabetes mellitus ^#^ | 13 (30.2) | 42 (9.4) | **< 0.001** |
| COPD ^#^ | 16 (37.2) | 87 (19.6) | **0.007** |
| Asthma | 0 (0.0) | 18 (4.0) | 0.357 |
| Chronic kidney disease | 5 (11.6) | 35 (7.9) | 0.570 |
| Solid malignant tumor ^#^ | 8 (18.6) | 27 (6.1) | **0.006** |
| Obesity (*n*, %) ^#^ | 11 (25.6) | 37 (8.3) | **0.001** |
| Smoking history (*n*, %) ^#^ | 18 (41.9) | 123 (27.6) | **0.049** |
| Baseline clinical and radiologic features (*n*, %) |  |  |  |
| Mental confusion ^#^ | 14 (32.6) | 65 (14.6) | **0.002** |
| Respiratory rates ≥ 30 beats/min ^#^ | 14 (32.6) | 41 (9.2) | **< 0.001** |
| SBP < 90 mmHg | 0 (0.0) | 4 (0.9) | 0.532 |
| Leukocytes > 10×10^9^/L | 13 (30.2) | 105 (23.6) | 0.332 |
| Lymphocytes < 0.8×10^9^/L ^#^ | 26 (60.5) | 38 (8.5) | **< 0.001** |
| HB < 100 g/L ^#^ | 14 (32.6) | 80 (18.0) | **0.021** |
| ALB < 30 g/L ^#^ | 21 (48.8) | 10 (2.2) | **< 0.001** |
| BUN > 7 mmol/L ^#^ | 23 (53.5) | 156 (35.1) | **0.017** |
| PO_2_/FiO_2_ < 250 mmHg ^#^ | 25 (58.1) | 115 (25.8) | **< 0.001** |
| Multilobar infiltrates | 39 (90.7) | 362 (81.3) | 0.132 |
| Pleural effusion | 7 (16.3) | 102 (22.9) | 0.318 |
| Coinfections (*n*, %) | 17 (39.5) | 132 (29.7) | 0.180 |
| Systemic corticosteroids use (*n*, %) ^#^ | 23 (53.5) | 23 (5.2) | **< 0.001** |

IQR: interquartile range; COPD: chronic obstructive pulmonary disease; SBP: systolic blood pressure; HB: haemoglobin; ALB: albumin; BUN: blood urea nitrogen; PO_2_/FiO_2_: arterial pressure of oxygen/fraction of inspiration oxygen. #: variables cited in the table above were the candidates which were entered into the multivariate logistic regression model. The bolded values are p-values < 0.05, which represented significant differences between deceased patients and survival patients.

Supplementary Figure 1 Distribution of patients with the three types of PMVs-p by months.
